# Analyzing the link between anxiety and eating behavior as a potential pathway to eating-related health outcomes

**DOI:** 10.1038/s41598-021-94279-1

**Published:** 2021-07-19

**Authors:** Felix S. Hussenoeder, Ines Conrad, Christoph Engel, Silke Zachariae, Samira Zeynalova, Heide Glaesmer, Andreas Hinz, Veronika Witte, Anke Tönjes, Markus Löffler, Michael Stumvoll, Arno Villringer, Steffi G. Riedel-Heller

**Affiliations:** 1 Institute of Social Medicine, Occupational Health and Public Health, University of Leipzig, Ph.-Rosenthal-Str. 55, 04103 Leipzig, Germany; 2 Institute for Medical Informatics, Statistics and Epidemiology (IMISE), University of Leipzig, Leipzig, Germany; 3 Department of Medical Psychology and Medical Sociology, University of Leipzig, Leipzig, Germany; 4 Max-Planck-Institute for Human Cognitive and Brain Sciences, Leipzig, Germany; 5 Medical Department III – Endocrinology, Nephrology, Rheumatology, University of Leipzig, Leipzig, Germany

**Keywords:** Public health, Psychology, Diseases, Risk factors

## Abstract

Anxiety is a widespread phenomenon that affects various behaviors. We want to analyze in how far anxiety is connected to eating behaviors since this is one potential pathway to understanding eating-related health outcomes like obesity or eating disorders. We used data from the population-based LIFE-Adult-Study (n = 5019) to analyze the connection between anxiety (GAD-7) and the three dimensions of eating behaviors (FEV)—Cognitive Restraint, Disinhibition, and Hunger—while controlling for sociodemographic variables, smoking, physical activity, personality, and social support. Multivariate regression analyses showed significant positive associations between anxiety and Disinhibition as well as Hunger, but not between anxiety and Cognitive Restraint. Interventions that help individuals to better regulate and cope with anxiety, could be one potential pathway to reducing eating disorders and obesity in the population.

## Introduction

Anxiety is a widespread phenomenon. For example, while data show that the lifetime prevalence of generalized anxiety disorder (GAD) is around 3.7%^1^, a review finds a lifetime prevalence of subthreshold GAD of around 12.4%^2^. A study of community-dwelling older adults (65+) found a 12 month prevalence of 26.2% for all subthreshold manifestations of anxiety^3^. While anxiety can be a mental health problem by itself, it is also connected to problematic eating-related health outcomes and behaviors. Anxiety has repeatedly been connected to obesity^4,5^ and to a variety of other eating disorders, i.e., anorexia nervosa^6,7^, bulimia nervosa^8,9^, and binge eating^10,11^ as well as to the sub-clinical forms of these disorders and weight concerns^12^. In the light of the strong empirical connection between anxiety and eating-related health outcomes our study is interested in how far anxiety is related to specific, detrimental eating behaviors. We assume that these behaviors play a crucial role in connecting anxiety on the one side with obesity and eating disorders on the other side. Furthermore, anxiety-related eating behaviors could also contribute to the connection between anxiety and other widespread diseases like cardiovascular disorder and diabetes that is reflected in meta-analyses^13,14^.

Identifying anxiety-related eating behaviors can contribute to the development of targeted interventions to combat obesity and other disorders and health problems in the population. There are already examples in the literature that connect anxiety with specific eating behaviors like selective eating in children^15^, disordered eating behaviors in adolescents^16^, unhealthy eating in university students^17^, and the increased consumption of saturated fats and added sugars^18^. If we also take into account the studies on eating behaviors in the context of eating disorders that we introduced above, it is clear that the focus of the literature is on inherently problematic—often highly specific—eating behaviors, and/ or young individuals. Since we are interested in a public health perspective and the broad applicability of our results, in our study, we want to shift focus to less clinical, broader eating behaviors and a more inclusive representation of the population. The three-factorial approach to eating behaviors was established in the 1980`s by Stunkard and Messick^19^ and has since then been applied around the globe and to a variety of research topics, from weight reduction and BMI to sleep and chrono type^20–23^. It refers to the following three domains of eating behavior: (1) Cognitive Restraint, i.e., the tendency of someone to restrict food consumption, a form of cognitive control that is associated with daily food intake; (2) disinhibition, i.e., an overconsumption of food due to a variety of stimuli and a loss of control with regard to food intake; and (3) Hunger, i.e., the susceptibility for internal or external hunger signs^19,24,25^.

The three-factors of eating behavior have already been linked to anxiety in recent studies. Janjetic et al.^26^ showed a relationship between all three factors and state anxiety in a sample of Argentinian women between 40 and 65 years, and Aoun et al.^27^ linked anxiety symptoms to uncontrolled eating, an amalgamation of the two factors Disinhibition and Hunger, in a sample of Lebanese university students. While these studies point towards a meaningful link between anxiety and the three factors of eating behavior, they were both conducted with highly specific samples from non-European countries. Since eating behaviors are embedded in and shaped by their cultural and social contexts, these results cannot easily be applied to a European context. Hence, the goal of this study is to analyze the connection between anxiety and all three factors of eating behavior in a European context and with a large sample that is broad and heterogeneous in terms demographics and social status.

## Methods

### Study design

The Adult Study of the Leipzig Research Centre for Civilization Diseases (LIFE) is a large population-based cohort study in the city of Leipzig, Germany, and a collaboration of several clinical and epidemiological research teams.

10,000 participants between 18 and 80 years were recruited through age- and gender-stratified random selection by the local residents’ registry office. The majority of participants were above 40, and the only exclusion criterion was being pregnant. The LIFE-Adult baseline examination took place between 2011 and 2014, and every participant provided written informed consent prior to participation. The participants underwent a set of assessments, including interviews, questionnaires, and medical examinations. Details on study design and assessments can be found elsewhere^28^.

### Ethics

The LIFE-Adult-Study complies with the ethical standards of the relevant national and institutional committees on human experimentation and with the Helsinki Declaration of 1975, as revised in 2008. The study was approved by the ethics committee of the University of Leipzig.

### Measures

#### Sociodemographic variables

Participants provided information on age, gender, medical history, marital status, smoking, and living situation in standardized interviews to trained study personnel. They also provided information on education, occupational status, and equivalent household income that was used to compute socioeconomic status (low, medium, high^29^).

#### Anxiety

We used the Generalized Anxiety Disorder Scale-7 (GAD-7^30,31^) to measure the level of anxiety. The GAD-7 contains seven items that can be answered on a scale from “0” (= never) to “3” (= almost every day). These items refer to typical anxiety symptoms, like worrying, nervousness, and irritability.

#### Eating behavior

We used the modified and extended German version of the three-factor-eating questionnaire (German: Fragebogen zum Essverhalten, FEV) to asses three factors of eating behavior^25,32^. The test is the German version of the Three-Factor-Eating-Questionnaire^19^, measuring Cognitive Restraint (21 items), Disinhibition (16 items), and Hunger (14 items). Items are in the form of statements like “I eat small portions on purpose because I do not want to gain weight” (Cognitive Restraint), “When I am sad, I often eat too much” (Disinhibition), or “Because I am hungry all the time, it is hard for me to stop eating before the dish is empty.” (Hunger).

#### Covariates

Participants completed the revised German version of the Ten Item Personality Inventory (TIPI^33^). The revised test comprises 16 items (scale range 1–7) assessing the Big Five personality traits, i.e. Neuroticism, Extraversion, Openness to Experience, Agreeableness, and Conscientiousness. Physical activity was assessed via the 7-item short form of the International Physical Activity Questionnaire (IPAQ-SF, https://sites.google.com/site/theipaq/), and social support via the 5-item ENRICHD Social Support Scale^34^. We selected personality, physical activity, and social support as covariates based on our own theoretical considerations as well as on the literature^35–37^.

### Statistical analyses

Statistical analyses were performed using IBM SPSS (Version 25). From the original sample of 10,000 participants we had to exclude participants who were living in retirement/nursing homes, with relatives or in some form of supported living because we assumed that this would affect their eating behaviors (N = 127). We also excluded people with diabetes (N = 1072), and those that were treated for diseases, when treatment or disease were likely to have an impact on eating behaviors, like ulcer or cancer (N = 1055). We then removed participants with missing values for GAD-7 (N = 332) FEV-scales (N = 1176/199/168), SES (N = 3), smoking (N = 75), IPAQ (N = 611), ESSI (N = 37), personality (N = 126). The persons that we removed due to missing values were significantly older, and more likely to be female and to exhibit a lower social economic status than the participants included in the final sample. The final sample contained 5019 participants.

We conducted linear regression analyses with anxiety as a predictor of (1) Cognitive Restraint, (2) Disinhibition, and (3) Hunger with and without sociodemographic variables, personality, physical activity, and social support as control variables. Before the regression analysis, we checked assumptions (linearity, homoscedasticity, multicollinearity, independence of residuals, normally distributed errors).

## Results

Our 5019 participants were 53.5 years old on average, and 51.6% were female. Table 1 gives an overview on the general characteristics of our sample.

**Table 1:** General characteristics of the study population.

	Total group (N = 5019)
Age	53.5 (11.7)
Gender (“female”)	2592 (51.6%)
**Marital status**	
Married, living with partner	2939 (58.6%)
Married, living separated	127 (2.5%)
Single	1113 (22.2%)
Divorced	680 (13.5%)
Widowed	160 (3.2%)
**Socioeconomic status**^**a**^	
Low	693 (13.8%)
Medium	3051 (60.8%)
High	1275 (25.4%)
**Personality (NEO16-AM; range 1–7)**	
Neuroticism	3.2 (1.1)
Extraversion	3.6 (1.2)
Openness	5.4 (0.9)
Agreeableness	5.9 (1.0)
Conscientiousness	5.9 (0.8)
Anxiety (GAD-7; 0–21)	3.3 (3.2)
Smoking (“yes”)	11.27 (22.5%)
**Physical activity (IPAQ)**	
Low	604 (12.0%)
Moderate	1459 (29.1%)
Intensive	2956 (58.9%)
Social support (ESSI; 5–25)	22.3 (3.5)
**Eating behavior (FEV)**	
Cognitive restraint (0–21)	7.4 (4.6)
Disinhibition (0–16)	4.2 (2.8)
Hunger (0–14)	3.4 (2.7)

Continuous variables are given as mean (standard deviation); categorical variables are displayed as numbers (percentages).

^a^Socioeconomic status was computed based on education, occupational status, and equivalent household income^29^.

Table 2 shows that anxiety positively predicted all three factors of eating behavior. Once control variables were added, anxiety still predicted Disinhibition and Hunger but no longer Cognitive Restraint. Male gender and smoking exhibited a negative and Neuroticism a positive relationship with all three dimensions of eating behavior. Age, conscientiousness, and intense physical activity all exhibited a positive association with Cognitive Restraint and a negative relationship with Disinhibition and Hunger. Socioeconomic status had a positive association with Cognitive Restraint and Disinhibition, Openness a positive one with Cognitive Restraint and a negative one with Hunger. In addition, Extraversion and Agreeableness both had a positive relationship with Cognitive Restraint, and Social Support a negative one with Disinhibition.

**Table 2:** Prediction of cognitive restraint, disinhibition, and hunger by anxiety (N = 5019; standardized regression coefficients).

	Cognitive Restraint		Disinhibition		Hunger	
Anxiety	0.04**	0.03	0.23***	0.15***	0.21***	0.14***
Age		0.17***		− 0.12***		− 0.11***
Gender^a^		− 0.22***		− 0.15***		− 0.07***
Marital status^b^		− 0.01		0.02		0.02
**Socioeconomic status**						
Medium		0.06**		0.05*		0.02
High		0.09***		0.09***		0.03
**Personality**						
Neuroticism		0.03*		0.04*		0.06***
Extraversion		0.03*		0.01		− 0.01
Openness		0.03*		− 0.01		− 0.03*
Agreeableness		0.06***		0.02		− 0.01
Conscientiousness		0.12***		− 0.15***		− 0.14***
Smoking (“yes”)		− 0.10***		− 0.07***		− 0.05***
**Physical activity (IPAQ)**						
Moderate		0.01		− 0.03		− 0.01
Intense		0.07***		− 0.06**		− 0.05*
Social support		− 0.02		− 0.05***		− 0.02
R^2^	0.00	0.16	0.05	0.13	0.04	0.10

*p ≤ 0.05; ^+^**p ≤ 0.01; ***p ≤ 0.001.

^a^Gender: 0 = female; 1 = male.

^b^Marital status: 0 = married, living with partner; 1 = married and living separated, single, divorced, widowed.

## Discussion

Our results showed a positive association between anxiety and all three domains of eating behavior. After controlling for sociodemographic variables, smoking, physical activity, personality, and social support, anxiety remained significantly associated with both Disinhibition and Hunger, but not Cognitive Restraint.

Cognitive Restraint refers to an individual`s cognitive effort that is targeted at reducing food intake and that involves elements like planning and decision making, e.g., in the form of a planned diet. Hence, Cognitive Restraint items address plans to eat small portions or to avoid specific foods, knowledge about calories, and attitudes towards dieting. Accordingly, some studies point in the direction that anxiety is connected to risk-avoidant decision making and higher rates of dieting^38,39^. The fact that there is no significant association between anxiety and Cognitive Restraint in our data may be explained by the inclusion of the Big Five personality traits, all of which exhibited significant associations with the outcome. It suggests that Cognitive Restraint represents a rather stable general tendency or predisposition that lacks the volatility and reactiveness to be affected by rather temporary fluctuations in anxiety, leading to a loss of significance of the association with anxiety once personality is included in the regression. This interpretation matches with similar results from a study on the effects of Neuroticism, Conscientiousness, and anxiety on Cognitive Restraint in morbidly obese patients^40^.

Unlike Cognitive Restraint, Disinhibition refers to an overconsumption of food in response to a variety of internal and external stimuli^19^, i.e., it represents a more volatile and reactive factor. Our results suggest that anxiety acts as an internal stimulus that increases Disinhibition and could therefore temporally counteract the more stable effects of Cognitive Restraint. It matches with research that connects anxiety to impulsive forms of food consumption like binge or night eating^10,11^ and that indicates that some forms of overeating that are a part of eating pathologies may function as a way to regulate and deal with negative emotions^41,42^. Disinhibition could therefore be a form of anxiety regulation or coping. Since overeating^43,44^ as well as binge eating^45,46^ have been linked to overweight and obesity, Disinhibition could be a valuable starting point for public health interventions targeting eating-related health outcomes. If further research strengthens the assumption of a causal effect of anxiety on Disinhibition, this could be the basis for interventions to promote alternative ways of anxiety regulation for individuals with subthreshold anxiety symptoms, ranging from physical activity and autogenic training to muscle relaxation and cognitive interventions.

While clinical research is pointing in the direction of a causal link from anxiety to eating behaviors and experimental studies show an effect of emotional state and anxiety on food intake^47,48^, we cannot rule out that this also goes in the opposite direction as other studies show effects of nutrition on anxiety^49,50^ and a connection between household food insecurity and anxiety^51^. Therefore, eating behaviors that trigger the consumption of specific foods could thereby affect anxiety levels. Future longitudinal research has to clarify in how far that holds true for Disinhibition.

Our results showed a significant positive relationship between anxiety and Hunger. This connection is supported by recent research on the neurobiological foundations of both phenomena. A current review shows that not only specific nuclei of the hypothalamus and extended amygdala are activated by both hunger and fear, but also that neuropeptides that are released during states of hunger can reduce anxiety^52^. From that perspective, Hunger could be seen as a form of subconscious, neurobiological anxiety regulation. The authors of the review conclude that neuropeptides released during states of satiety could increase anxiety, which could additionally reinforce hunger as a form of emotion regulation. Hence, individuals would experience both, elevated states of hunger as well as anxiety, at different times which matches with our results. Future research needs to assess both variables in a synchronic way and over a longer period of time to strengthen our interpretation of the results.

This study has several advantages, e.g., multiple control variables, and the exclusion of participants with diseases/treatments that could affect the outcomes. In addition, we used a large and diverse sample from a German cohort study, and therefore our results can be generalized to some extent to a German and even Western European level. However, there are also certain limitations. First, future research needs to apply different and longitudinal research designs in order to further strengthen causal interpretations. Second, we removed a large part of the original sample due to missing values, resulting in a younger sample with a higher socioeconomic status and a more balanced distribution of gender. While this could be seen as a limitation of our study, it is not necessarily limiting the generalizability of our results since our final sample is even closer to the general population in terms of age and gender.

## Data Availability

The data that support the findings of this study are available from the corresponding author upon reasonable request.
